# Computational identification of novel natural inhibitors of glucagon receptor for checking type II diabetes mellitus

**DOI:** 10.1186/1471-2105-15-S16-S13

**Published:** 2014-12-08

**Authors:** Sonam Grover, Jaspreet Kaur Dhanjal, Sukriti Goyal, Abhinav Grover, Durai Sundar

**Affiliations:** 1 Department of Biochemical Engineering and Biotechnology, Indian Institute of Technology (IIT), Delhi, Hauz Khas, New Delhi, 110016, India; 2 Apaji Institute of Mathematics & Applied Computer Technology, Banasthali University, Tonk, Rajasthan, 304022, India; 3 School of Biotechnology, Jawaharlal Nehru University, New Delhi, 110067, India

**Keywords:** Glucagon Receptor, 7 transmembrane Domain, Natural Inhibitor, Docking, Virtual Screening

## Abstract

**Background:**

Interaction of the small peptide hormone glucagon with glucagon receptor (GCGR) stimulates the release of glucose from the hepatic cells during fasting; hence GCGR performs a significant function in glucose homeostasis. Inhibiting the interaction between glucagon and its receptor has been reported to control hepatic glucose overproduction and thus GCGR has evolved as an attractive therapeutic target for the treatment of type II diabetes mellitus.

**Results:**

In the present study, a large library of natural compounds was screened against 7 transmembrane domain of GCGR to identify novel therapeutic molecules that can inhibit the binding of glucagon with GCGR. Molecular dynamics simulations were performed to study the dynamic behaviour of the docked complexes and the molecular interactions between the screened compounds and the ligand binding residues of GCGR were analysed in detail. The top scoring compounds were also compared with already documented GCGR inhibitors- MK-0893 and LY2409021 for their binding affinity and other ADME properties. Finally, we have reported two natural drug like compounds PIB and CAA which showed good binding affinity for GCGR and are potent inhibitor of its functional activity.

**Conclusion:**

This study contributes evidence for application of these compounds as prospective small ligand molecules against type II diabetes. Novel natural drug like inhibitors against the 7 transmembrane domain of GCGR have been identified which showed high binding affinity and potent inhibition of GCGR

## Background

Diabetes mellitus comprises a group of metabolic diseases which are rapidly growing worldwide. It has so far affected approximately 347 million people globally [1]. Glucagon receptor (GCGR) is an affiliate of secretin-like (class B) family of G-protein-coupled receptors (GPCRs) in humans [2]. Secretin-like GPCRs contain a globular N-terminal extracellular domain (ECD) defined by three conserved disulphide bonds [3,4] and a 7 transmembrane (7 TM) domain. GCGR is activated by a 29 amino acid long peptide hormone, Glucagon, which is secreted by pancreatic α-cells in response to decreased circulating blood glucose levels. GCGR helps in maintaining glucose homeostasis by increasing hepatic gluconeogenesis and glycogenolysis [5]. Binding of glucagon to GCGR activates signal transduction pathway leading to the activation of adenylate cyclase. This triggers the production of cAMP which activates the protein kinase A, that finally results in an increase in blood glucose levels [6]. In type 2 diabetes mellitus, increase in the level of glucagon secretion takes place in both the fasting and postprandial state caused due to either impaired pancreatic α-cell sensing, or lack of suitable α-cell response to insulin [5]. It has been reported that glucagon receptor knockout in mice prevents the deadly metabolic and clinical phenotype of type 1 diabetes [7]. The inhibition of glucagon-GCGR interaction has been reported to control the hepatic glucose overproduction that makes it an attractive therapeutic strategy for the treatment of type II diabetes mellitus.

Most of the available glucagon receptor based inhibitors for the treatment of type 2 diabetes mellitus fall in the category of glucagon neutralizing antibodies [8,9] or small molecular weight glucagon receptor antagonists [10-15] which have been shown to efficiently terminate glucagon receptor action. A new glucagon receptor antisense oligonucleotides was developed as potential therapeutic agent for type 2 diabetes mellitus [16]. In spite of the above advances, there are concerns corresponding to safety, tolerability and stimulation of adverse immune responses with the above mentioned agents to reduce glucagon receptor signalling. In view of these concerns, glucagon receptor antagonists of natural origin may offer a favourable therapeutic option helping the patients attain a proper glycemic control and to evade the long-standing obstacles related with this disease.

Due to the lack of crystal structure of class B 7 TM domains, discovery of clinically functional small molecule glucagon receptor antagonists was difficult. Till date few GCGR-ligand binding models have been proposed which are based on the approach of site-directed mutagenesis [17-19], photo-crosslinking [20-22], and modelled structure-based virtual screening studies [23]. However with the recent elucidation of the crystal structure of 7 TM domain of glucagon receptor, a rational drug design approach can be applied for identification of potent agents against type 2 diabetes [24].

In the present study, we have identified novel natural GCGR antagonists based on the GCGR 7 TM domain crystal structure. The inhibition of glucagon receptor results in overall glycemic control and improved glucose tolerance. A large virtual database of natural compounds was screened against the high resolution crystal structure of GCGR using high throughput virtual screening approach. *In silico *screening led to the identification of a new class of GCGR inhibitors that hinder the GCGR-glucagon interaction. The molecular dynamics (MD) of the complexes were then simulated to elucidate the dynamic behaviour of molecular interactions between the screened compounds and the functional residues of GCGR. This study smoothens the path for the development of novel leads possessing improved binding properties, high drug likeness and low toxicity to humans for type 2 diabetes mellitus treatment.

## Methods

### Protein and ligand library preparation

The crystal structure of human glucagon receptor [PDB ID: 4L6R], determined at a resolution of 3.40 Å, was retrieved from the Protein Data Bank [24]. GCGR contains a 7 TM helical domain. The retrieved structure was processed using the Protein Preparation Wizard in Schrodinger's Maestro interface [25] to prepare it for docking studies. It involved addition and optimization of hydrogen bonds, removal of bad contacts, optimization of bond lengths, creation of disulfide bonds, capping of protein terminals, conversion of selenomethionine to methionine and fixing of missing residues. The prepared structure was then optimized to acquire an energetically stable geometry [26]. A virtual ligand library was prepared by extracting proximately 1,69,109 natural compounds from the ZINC database [27] and processing them with Schrodinger's LigPrep Wizard [28]. Further, maximum possible tautomeric, stereochemical and ionization variants of these molecules were generated.

### Grid generation, high throughput virtual screening and extra precision docking studies

As suggested by GCGR-glucagon binding model, Trp 36, Gln 142, Tyr 138, Tyr 149, Val 191, Gln 232, Glu 362, Leu 386, Trp 295 and Asn 298 residues of GCGR are directly involved in binding with glucagon [20]. A grid was generated in the region of these functional residues of prepared protein by means of the receptor grid generation utility of the Glide docking module of the Schrodinger suite [29,30]. For screening of prepared libraries Glide program was applied [29,31]. Glide algorithm is derived from a systematic method for virtual screening with incremental construction searching and provides the output as the G-Score scoring function combined with a range of other parameters [32]. The screening against 7 TM domain of GCGR at the essential grid coordinates was initially performed with the HTVS docking algorithm [29]. Compounds with a significant docking score were then, subjected to Glide XP, a more accurate docking algorithm for further refined screening [28].

### Molecular dynamics simulations of docked complexes

In order to examine the stability of top scoring compounds molecular dynamics simulations were performed using Desmond Molecular Dynamic System [33] with Optimized Potentials for Liquid Simulations (OPLS) all-atom force field 2005 [34]. The protein-ligand complexes obtained from Glide's XP docking protocol were prepared using Desmond set-up wizard. All the missing residues were rectified manually. The system, thus obtained was solvated in a triclinical periodic box of SPC water and neutralized with suitable number of counter-ions. The distance between box wall and protein-ligand complex was set to greater than 10 Å so that the complex does not directly interact with its own periodic image. Energy minimization of the prepared system was done up to a maximum of 10 steps using steepest descent method or until a gradient threshold (25 kcal/mol/Å) was obtained. Desmond's default protocol was applied to equilibrate the system. The details of the relaxation protocol used for equilibration and minimization steps are given in additional file 1. MD simulations were performed on this equilibrated system for a time period of 20 ns at 300 K constant temperature and 1atm constant pressure using a time step of 2 fs. Throughout the simulations process, smooth particle Mesh-Ewald method was used to calculate long range electrostatic interactions. A 9 Å radius cut-off was set for coulombic short range interaction cutoff method. At every 4.8 ps time step, frames were captured to form trajectory. The configuration file used for simulation process is given in additional file 2.

The root mean square deviation (RMSD) of the protein-ligand complex was calculated for the entire simulations trajectory in reference to the first frame. Ligplot program was used for the calculation of hydrogen bonds and other non-bonded interactions [35].

### Comparison of the proposed natural compounds with some already documented GCGR antagonists

Several compounds have been developed by various groups with inhibitory activity against glucagon receptor for the treatment of type II diabetes mellitus. Recently, MK-0893 (Merck) [36] and LY2409021 (Eli Lilly) [37] have been reported with GCGR inhibitory activity supported with clinical data. The 3D structure of MK-0893 was retrieved from PubChem database. The structure of LY2409021 was prepared in 3D sdf format using Marvin sketch. These two compounds were further prepared using LigPrep to look for different possible conformations. The compounds were then docked against the GCGR structure using the same grid coordinates generated around the catalytically active residues of the GCGR receptor.

### Prediction of pharmacokinetics of CAA and PIB in human body and comparison of the results with the already known antagonists

Certain structural and molecular features of compounds govern their pharmacokinetic properties in our body. Qikprop module of Schrodinger [38] was used to evaluated the drug likeliness of all the four inhibitors. The obtained values for molecular weight, number of hydrogen bond donors, number of hydrogen bond acceptors and logP were used to assess violation of Lipinski's rule of five if any. To further account for the potential of the compounds to act as efficient drug candidates, their absorption, distribution, metabolism and excretion (ADME) properties were also calculated *in silico *using Qikprop.

## Results and discussion

### Outcomes of high throughput virtual screening and docking studies

**Figure 1:**
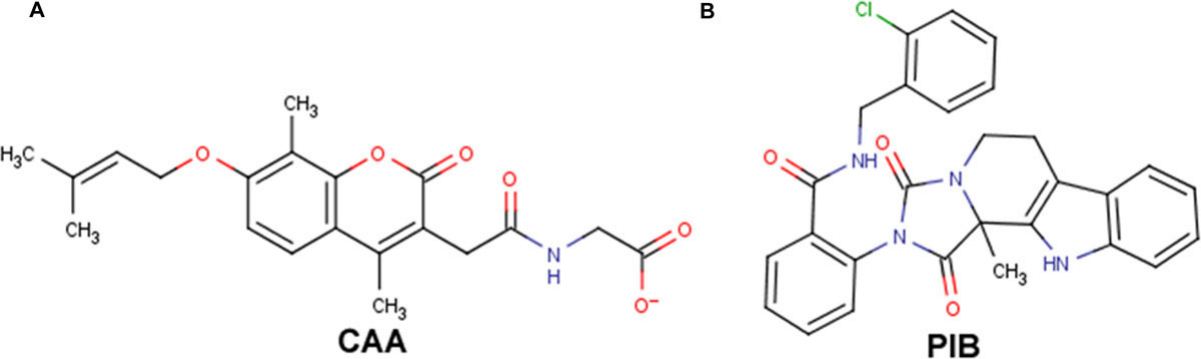
**Chemical structure of (A) CAA and (B) PIB**.

Human GCGR, one of the most promising drug targets for treatment of type II diabetes mellitus, was virtually screened against approximately 0.2 million natural compounds, a special subset of ZINC database. The screened compounds were ranked according to their binding affinity, calculated as the scoring function called the GlideGScore. Of all the compounds that were identified from HTVS, those with a Glide score of less than −6.0 (64 compounds) were subjected to the Glide XP docking protocol. Among the top 10 scoring compounds 2 compounds which were fulfilling the Lipinski's filter criteria were chosen for further studies and their properties are listed in Table 1. The values of the other important docking parameters like ligand efficiency score, glide emodel score etc. used for evaluating the selection criteria of the top-scoring ligands are shown in Table 2. The top-scoring compound *(S)-N-(2-chlorobenzyl)-2-(11b-methyl-1,3-dioxo-5,6-dihydro-1H-imidazo[1',5':1,2]pyrido[3,4-b]indol-2(3H,11H,11bH)-yl)benzamide *(*ZINC12864028*; PIB) had a Glide score of −9.47 kcal/mol, while the second compound *2-(2-(4,8-dimethyl-7-((3-methylbut-2-en-1-yl)oxy)-2-oxo-2H-chromen-3-yl) acetamido)acetic acid *(*ZINC06623951;*CAA) had a score of −9.53 kcal/mol. Both the compounds had good binding affinity for the GCGR receptor. Their chemical structure is given in Figure 1. The results revealed that both the compounds, PIB and CAA, were interacting with the important glucagon binding residues of GCGR by hydrogen bond and hydrophobic interactions (Table 3).

**Table 1:** Values of various physico-chemical descriptors.

Descriptors	CAA	PIB	MK-0893	LY2409021	Recommended range
**#star**	0	0	4	0	0-5

**Molecular weight**	373.405	498.968	588.489	294.35	<500

**Hydrogen bond donors**	1.25	1	4.25	1	<5

**Hydrogen bond acceptors**	7	5	5.25	5.5	<10

**logP**	2.618	5.485	8.18	-1.062	<5

**SASA**	707.226	652.412	900.01	594.833	300-1000

**Oral absorption**	64.462	100	85.123	28.784	>80% high <25% poor

**CNS activity**	0	-2	-2	-2	-2 inactive 2 active

**Blood brain barrier partition coefficient**	-2.063	-0.317	-1.179	-1.8	-3.0-1.2

**Table 2:** Binding affinity scores and energies of GCGR in complex with PIB and CAA.

Compound	Zinc ID	Docking Score	Glide Ligand Efficiency	Glide evdw (kcal/mol)	Glide emodel	Glide energy (kcal/mol)
PIB	ZINC06623951	-9.53	-1.06	-31.69	-52.56	-39.41

CAA	ZINC12864028	-9.47	-0.87	-35.75	-54.20	-38.82

MK-0893	-	-11.03	-0.27	-34.77	-67.70	-46.01

Ly2409021	-	-5.61	-0.27	-24.37	-32.40	-32.24

**Table 3:** Molecular interactions displayed in pre- and post-MD simulated PIB-bound GCGR complexes.

GCGR-Ligand	Residues involved in H-bond formation	H-Bond Length (Å)	Residues involve in hydrophobic interactions
PIB Pre-MD	Tyr145 Trp295	3.30 3.08	Gln232, Thr296, Asn298, Phe365, Met231, Leu307, Ile235, Phe383, Phe303, Gln142, Leu386, Asp195, Val191, Ser389, Tyr149

PIB Post-MD	Val364	2.88	Glu346, Ser389, Val363, Tyr149, Tyr145, Phe365, Leu382, Gln142, Gln232, Gln231, Trp295, Phe303, Leu386, Phe383

CAA Pre-MD	Ser389, Gln392	3.19, 2.54 3.15	Lys187, Leu386, Gln293, Met231, Glu362, Phe365, Trp295, Ile235 Leu198, Tyr149, Tyr145, Val191, Ile194

CAA Post-MD	Ser389	2.89	Lys187, Tyr133, Trp295, Leu370, Gln142, Ile235, Val191, Leu198, Leu382, Met231, Ile194, Gly362, Asp385

### Binding mode analysis of ligand-docked GCGR complexes

#### GCGR-PIB Complex

**Figure 2:**
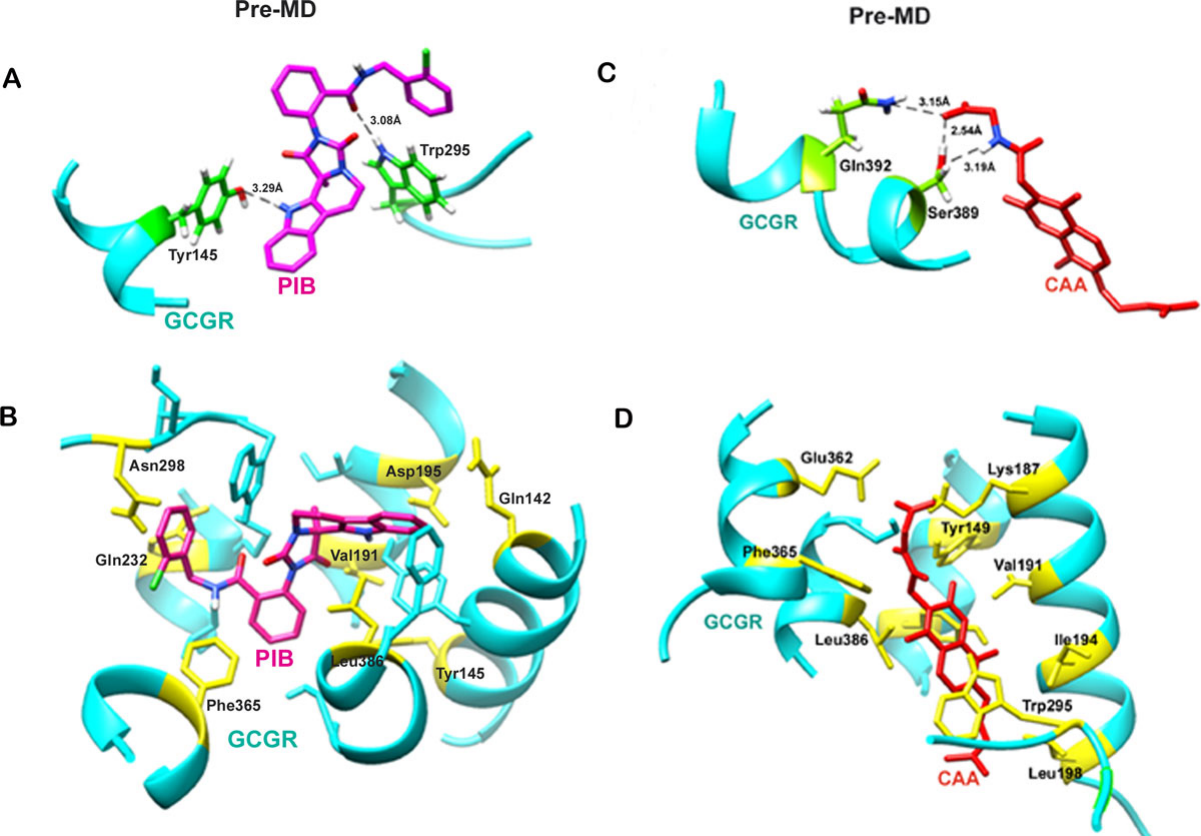
**Molecular interactions between GCGR and screened ligands before MD simulations**. (A) Pre-MD Hydrogen bond interactions in GCGR-PIB complex. (B) Pre-MD Hydrophobic interactions between GCGR and PIB (C) Pre-MD Hydrogen bond interactions in GCGR-CAA complex. (D) Post-MD Hydrophobic interactions between GCGR and CAA.

GCGR is a class B G protein coupled receptor where 7 TM helices create a deep and wide cavity for ligand binding. In the case of the GCGR-PIB complex, PIB interacted with the glucagon binding residues of GCGR with the formation of 2 hydrogen bonds and a number of hydrophobic and van der Waal contacts. Tyr145 and Trp295 were the two residues involved in hydrogen bond formation (Figure 2A). The H atom of OH group of amino acid Tyr145 was forming hydrogen bond (3.28 Å) with the N1 atom of PIB. Second hydrogen bond (3.08 Å) was formed by atom O3 of PIB with the NE1 atom of GCGR residue Trp295. In addition, Gln232, Asn298, Phe365, Gln142, Leu386, Asp195, Val191 and Tyr149 residues of GCGR were involved in hydrophobic and van der Waal interactions in the GCGR-PIB complex (Figure 2B). Among all these interacting residues, Trp295, Asn298 and Gln142 have been shown to directly interact with glucagon while mutation in Tyr145, Asp195 and Leu386 has been shown to decrease the glucagon binding. Tyr149, Gln232, Val191 and Glu109 are among the other residues that line the binding pocket of the GCGR. These are all crucial amino acids and play a prominent role in binding of glucagon with GCGR. PIB binding to these residues can thus prevent interaction between glucagon and GCGR thereby inhibiting the downstream signal transduction pathway. A variety of physicochemical properties of PIB were also considered which corroborate its drug like characteristics (Table 1).

#### GCGR-CAA complex

CAA is a small compound with a molecular weight of 372.29 g/mol and lipophilicity value (logP) of 2.618 at pH 7. CAA formed 3 hydrogen bonds and numerous hydrophobic interactions with human GCGR. As illustrated in Figure 2C, 2 hydrogen bonds were formed between the OG atom of Ser389 and the O6 & N1 atom of CAA with bond length 2.54Å and 3.19Å, respectively, while one more bond was formed with the NE2 atom of the neighbouring residue Gln392 and O6 atoms of CAA with bond length of 3.15 Å. However, several residues like Lys187, Leu386, Glu362, Phe365, Trp295, Leu198, Tyr149, Tyr145, Val191, and Ile194 important for ligand binding were involved in making hydrophobic and van der Waal contacts with CAA (Figure 2D). These interactions of CAA with the crucial functional residues of GCGR suggest it to be a promising ligand that could abolish the binding of glucagon with GCGR.

### Molecular dynamics simulations of ligand-bound GCGR complexes

For further refinement and stabilization of both the docked complexes, molecular dynamics simulation was carried out for GCGR in complex with the compounds for 20 ns using Desmond. The simulation length of 20 ns used in the study was sufficient enough to permit reorganization of the side chains of ligand-complexed protein thereby allowing it to acquire the energetically most stable binding conformation.

### Interaction analysis of MD-stabilized GCGR-PIB complex

**Figure 3:**
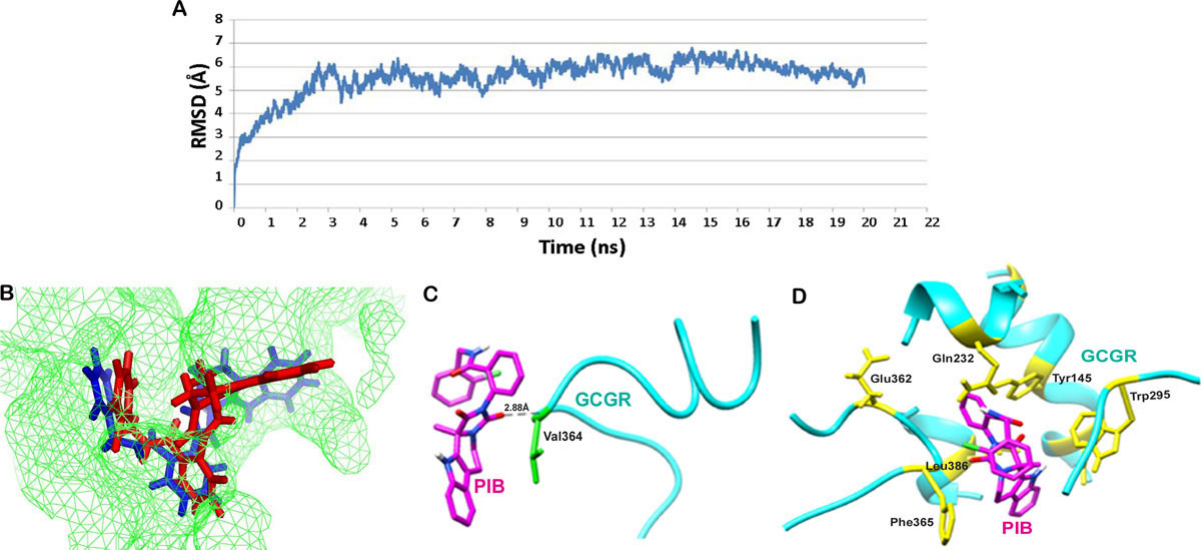
**(A) RMSD trajectory of GCGR in complex with PIB over the 20 ns simulation run**. (B) Change in orientation of PIB after simulation (red-post MD, blue-pre MD). (C) Hydrogen bond interactions between PIB and GCGR after MD simulations. (D) Hydrophobic contacts found in GCGR-PIB complex post MD simulation.

To study the dynamic nature of interactions, molecular dynamics simulation was carried out for GCGR in complex with PIB for 20 ns. The frames were captured after every 4.8 ps during the simulation run. RMSD of the protein backbone in each frame in reference to the first frame was plotted against the simulation run time. The backbone of the protein in the complex deviated upto 6 Å in the first 3 ns after which it acquired an almost stable conformation which then persisted till the end of the simulation period (Figure 3A). A structure was obtained by averaging the coordinates of all the frames in the most stable time frame, which was then used for further analysis. Even though a slight shift was observed in the binding mode of PIB, it was still occupying the cavity found within the 7TM domain of GCGR, the active cleft where glucagon interacts with GCGR. The superimposition of the ligand PIB in the pre- and post-MD simulated complex structures inside the ligand binding site of GCGR is depicted in Figure 3B. A comparative analysis of the interaction profiles of GCGR-PIB complex before and after the MD simulations is described in Table 3. Because of the change in the orientation of PIB, a difference was observed in binding pattern with GCGR. The 2 hydrogen bonds with the residues Tyr145 and Trp295 formed subsequent to docking were lost and a new hydrogen bond with amino acid Val364 came into existence (Figure 3C). Val364 is a conserved residue found in all class B GPCR's and is also involved in ligand binding [39]. Post simulation, PIB was found forming hydrophobic interactions and van der Waal contacts with various residues as mentioned in Table 3 while interaction with only those residues participating in glucagon binding are shown in Figure 3D.

### Interaction analysis of the GCGR-CAA complex post MD simulation

**Figure 4:**
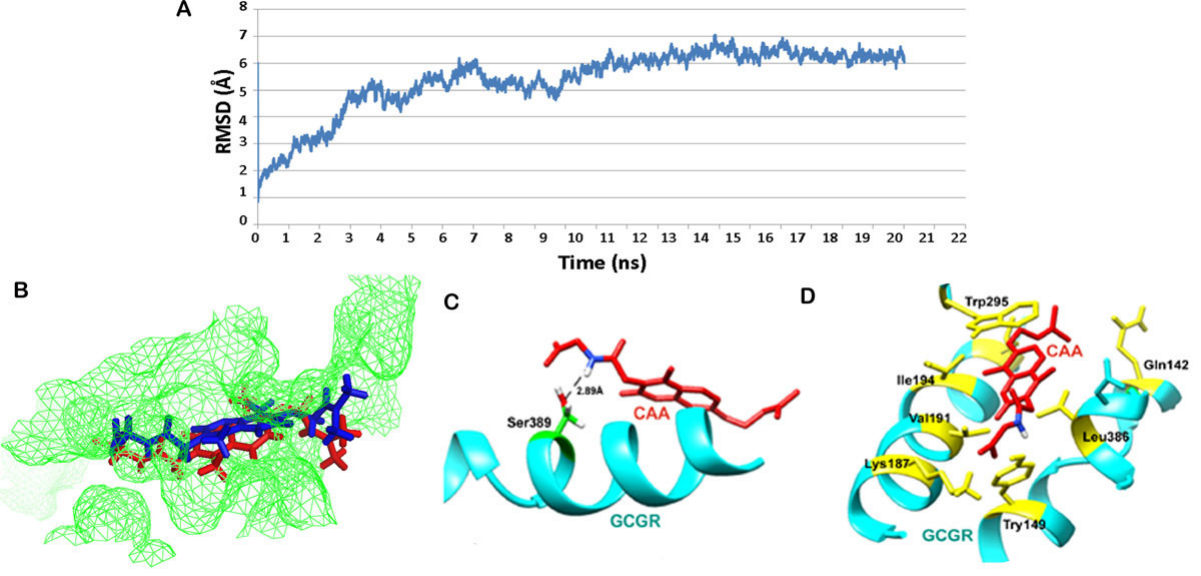
**(A) RMSD trajectory of GCGR protein backbone when complexed with CAA over the 20 ns simulation run**. (B) Position of CAA in GCGR active cavity before and after simulation (red-post MD, blue-pre MD). (C) Hydrogen bond interactions between CAA and GCGR after MD simulations. (D) Hydrophobic interactions found in GCGR-CAA complex post MD simulation.

Again to mimic the bodily conditions, a 20 ns long MD simulation was carried out for GCGR in complex with CAA. The backbone of protein in complex deviated about 6 Å in the first 10 ns after which a stable trajectory was observed. The newly attained conformation was more stable and thus persisted till the end of simulation without undergoing considerable change (Figure 4A). A shift was observed in the position of the docked ligand. The complexes obtained after molecular docking and MD simulation were superimposed to perceive the deviation in the conformation of CAA (Figure 4B). An average representative structure of complex was computed for the most stable time period (14-20 ns) of the simulation to study the molecular interaction pattern. A significant change in the H-bond interaction pattern was observed during the simulation run. Only the H-bond formed by CAA with Ser 389 of GCGR remained conserved (Figure 4C). The residues of GCGR which were involved in other non-bonded interactions with CAA now included Lys187, Tyr133, Trp295, Leu370, Gln142, Ile235, Val191, Leu198, Leu382, Met231, Ile194, Gly362 and Asp385 (Figure 4D). Many of these residues are involved in direct binding with glucagon or indirectly affect the conformation of ligand binding pocket. Interaction with these residues will restrict the binding of glucagon with GCGR and down modulate the functionality of the receptor by deterring the downstream signalling process. Therefore, we propose CAA as another prospective candidate for GCGR inhibition.

### Comparison of CAA and PIB with already documented antagonists of GCGR

The Glide docking score of MK-0893 and LY2409021 was compared with that of the proposed lead candidates. Table 2 lists the glide XP docking score of CAA, PIB and these two known GCGR inhibitors used in this study. MK-0893 with a score of -11.03 showed much higher affinity for GCGR in comparison to CAA and PIB whereas LY2409021 came out to be a weak candidate with much lower binding affinity (glide docking score = -5.60). The other glide parameters also followed the similar trend (table 2), indicating MK-0893 to be the best binder followed by PIB, CAA and then Ly2409021. Other physico-chemical properties of these compounds were also studied to further explore the potential of these compounds as drug candidates.

### Physico-chemical properties and pharmacokinetics of GCGR inhibitors- CAA, PIB, MK-0893, LY2409021

To supplement the information gained from binding affinity prediction, Qikprop was used to calculate various other physically significant descriptors and pharmaceutically relevant properties of these small molecules. Qikprop predicts these molecular properties and provides significant ranges for comparing their values with those of 95% of already known pharmaceutical drugs. The descriptor, "#star" denotes the number of outlying properties of the molecule i.e., the properties which do not fall within the range of values for already known drugs. So, lesser the number better is the druglikeness of the small molecule. MK-0893, which showed the highest binding affinity, had a #star value of 4 whereas all the other three compounds had 0 #star. Hence, except for MK-0893, the computed properties for the other three compounds did not lie outside the required range and were very similar to that of the known drugs. Lipinski's rule of five is a thumb rule which determines the likeliness of a drug to be orally active based on four molecular properties. Table 1 lists the values of all four properties for these four compounds. MK-0893 with a molecular weight of 588.48 and logP value of 8.18 was not satisfying the lipinski's rule (molecular weight < 500, no. of hydrogen bond donors < 5, no. of hydrogen bond acceptors < 10, logP < 5). Solvent accessible surface area (SASA) and especially polar surface area (PSA) dictate the passive transport of molecules through membranes thereby giving an estimate about the transport properties of the drugs. The total SASA for MK-0893, CAA, PIB and LY2409021 was well within the range given by QikProp. Using some knowledge based set of rules Qikprop also calculates the percentage probability of the drug getting orally absorbed in the human body. This value has been shown to correlate well with the human oral absorption. PIB showed the highest oral absorption with a percentage value of 100%. Out of rest three, LY2409021 had the least value of 28.78 %. Central nervous system activity is another parameter that needs to be considered for assessing the safety issue. CAA was found to be highly CNS inactive whereas PIB was predicted to possess some minimal amount of CNS activity. Blood brain barrier (BBB) separates the human brain from the direct contact of circulatory system, thus protecting the brain for unwanted solute particles. Both the predicted compounds were shown to be BBB negative ensuring their administration safe for the brain.

Even though MK-0893 had higher affinity for GCGR, CAA and PIB were found to be better than this known inhibitor in many aspects. The new compounds showed better druglikeness with acceptable values of ADME properties. This clearly delineates the distinctive potential of CAA and PIB as prospective lead inhibitors of GCGR for the treatment of type II diabetes mellitus.

## Conclusion

Human glucagon receptor has an important role in glucose homeostasis. Its activity can be regulated to treat type II diabetes mellitus. Some of the known GCGR antagonists have been found to be lipophilic molecules, with relatively high molecular weight, making them unfit for clinical use. With the intention to develop safe compounds with acceptable biopharmaceutical properties, we have proposed two novel inhibitors of natural origin for human glucagon receptor. This study provides evidence for consideration of these compounds as prospective small ligand molecules for inhibition of glucagon receptor.

## Competing interests

The authors declare that they have no competing interests.

## Authors' contributions

SoG designed the methods and experimental setup. SoG and JKD carried out the implementation of the various methods and were assisted by SG. SoG, AG and DS wrote the manuscript. All authors have read and approved the final manuscript.

## Supplementary Material

Additional file 1 **Relaxation protocol used for equilibration and minimization step**. Click here for file

Additional file 2 **Configuration file used for molecular dynamics simulation runs**. Click here for file

## Declaration

Funding for open access charges: IIT Delhi (IRD/RP02817 to D.S.)

This article has been published as part of *BMC Bioinformatics *Volume 15 Supplement 16, 2014: Thirteenth International Conference on Bioinformatics (InCoB2014): Bioinformatics. The full contents of the supplement are available online at http://www.biomedcentral.com/bmcbioinformatics/supplements/15/S16.
