# Supplementary material for: Computational identification of novel natural inhibitors of glucagon receptor for checking type II diabetes mellitus
Source: BMC Bioinformatics. 2014 Dec 8;15(Suppl 16):S13. doi: 10.1186/1471-2105-15-S16-S13 (PMC4290642; doi:10.1186/1471-2105-15-S16-S13)
Supplement: Additional file 1 — Relaxation protocol used for equilibration and minimization step. [file 1471-2105-15-S16-S13-S1.docx]

**Additional file 1-** Relaxation protocol used for equilibration and minimization step.

# Desmond standard NPT relaxation protocol

# All times are in the unit of ps.

# Energy is in the unit of kcal/mol.

task {

task = "desmond:auto"

set_family = {

desmond = {

checkpt.write_last_step = no

}

}

}

minimize {

title = "Minimization with restraints on solute"

max_steps = 2000

steepest_descent_steps = 10

convergence = 50.0

restrain = { atom = solute force_constant = 50.0 }

}

minimize {

title = "Minimization without any restraints"

max_steps = 2000

steepest_descent_steps = 10

convergence = 5.0

}

simulate {

title = "Berendsen NVT, T = 10 K, small timesteps, and restraints on solute heavy atoms"

annealing = off

time = 12

timestep = [0.001 0.001 0.003]

temperature = 10.0

restrain = { atom = solute_heavy_atom force_constant = 50.0 }

ensemble = {

class = NVT

method = Berendsen

thermostat.tau = 0.1

}

randomize_velocity.interval = 1.0

eneseq.interval = 0.3

}

simulate {

title = "Berendsen NPT, T = 10 K, and restraints on solute heavy atoms"

annealing = off

time = 12

temperature = 10.0

restrain = retain

ensemble = {

class = NPT

method = Berendsen

thermostat.tau = 0.1

barostat .tau = 50.0

}

randomize_velocity.interval = 1.0

eneseq.interval = 0.3

}

solvate_pocket {

should_skip = true

}

simulate {

title = "Berendsen NPT and restraints on solute heavy atoms"

effect_if = [["@*.*.annealing"] 'annealing = off temperature = "@*.*.temperature[0][0]"']

time = 12

restrain = retain

ensemble = {

class = NPT

method = Berendsen

thermostat.tau = 0.1

barostat .tau = 50.0

}

randomize_velocity.interval = 1.0

eneseq.interval = 0.3

}

simulate {

title = "Berendsen NPT and no restraints"

effect_if = [["@*.*.annealing"] 'annealing = off temperature = "@*.*.temperature[0][0]"']

time = 24

ensemble = {

class = NPT

method = Berendsen

thermostat.tau = 0.1

barostat .tau = 2.0

}

eneseq.interval = 0.3

}

simulate {

cfg_file = "configuration_file_name.cfg"

jobname = "$MASTERJOBNAME"

dir = "."

compress = ""

}
