# Supplementary material for: Computational identification of novel natural inhibitors of glucagon receptor for checking type II diabetes mellitus
Source: BMC Bioinformatics. 2014 Dec 8;15(Suppl 16):S13. doi: 10.1186/1471-2105-15-S16-S13 (PMC4290642; doi:10.1186/1471-2105-15-S16-S13)
Supplement: Additional file 2 — Configuration file used for molecular dynamics simulation runs. [file 1471-2105-15-S16-S13-S2.docx]

**Additional file 2-** Configuration file used for molecular dynamics simulation runs.

annealing = false

backend = {

}

checkpt = {

first = 0.0

interval = 240.0

name = "$JOBNAME.cpt"

write_last_step = true

}

coulomb_method = ["pme" 1e-09 ]

cpu = [2 2 5 ]

cutoff_radius = 9.0

elapsed_time = 0.0

energy_group = false

eneseq = {

first = 0.0

interval = 1.2

name = "$JOBNAME$[_replica$REPLICA$].ene"

}

ensemble = {

barostat = {

tau = 2.0

}

class = "NPT"

method = "MTK"

thermostat = {

tau = 1.0

}

}

fep = {

i_window = ?

lambda = "default:12"

output = {

first = 0.0

interval = 1.2

name = "$JOBNAME$[_replica$REPLICA$].dE"

}

}

glue = "solute"

maeff_output = {

first = 0.0

interval = 120.0

name = "$JOBNAME$[_replica$REPLICA$]-out.cms"

trjidx = "$JOBNAME$[_replica$REPLICA$]-out.idx"

}

meta = false

pressure = [1.01325 "isotropic" ]

randomize_velocity = {

first = 0.0

interval = inf

seed = 2007

temperature = "@*.temperature"

}

simbox = {

first = 0.0

interval = 1.2

name = "$JOBNAME$[_replica$REPLICA$]_simbox.dat"

}

surface_tension = 4000.0

taper = false

temperature = [

[300.0 0 ]

]

time = 20000.0

timestep = [0.002 0.002 0.006 ]

trajectory = {

first = 0.0

frames_per_file = 25

interval = 4.8

name = "$JOBNAME$[_replica$REPLICA$]_trj"

periodicfix = true

write_velocity = false

}
